# An Unusual Opportunity

**Published:** 1887-11

**Authors:** 


### An Unusual Opportunity.

The readers of the REGISTER will remember that in the March number of the present volume of the REGISTER, page 158, some note was made of a very valuable work just issued by Professor C. H. Stowell of the University of Michigan. As to the value of the work we do not wish to add much to what was then said, but can now fully emphasize all that was then said, being more familiar with the work than at that time. We are more than ever convinced of the value of the work to every dentist who wishes to understand the true structure of the various parts of the teeth. A good understanding of these tissues can be more easily obtained through this work than from any other with which we are acquainted, and certainly more readily than through some of the more pretentious works. The illustrations in this have probably never been equalled for accuracy of drawing, fulness of detail, and correctness of presentation. The working diagram alone—plate 2—is worth more than the price of the book to any student of dentology.

Arrangements have been made by which every subscriber to the REGISTER can have a copy of this work at a mere nominal price, for which see the advertising pages of this number.

The number of copies available under this arrangement is limited, and the first applicants will be first served.

